# Soluble Sema4D Level Is Positively Correlated with Sema4D Expression in PBMCs and Peripheral Blast Number in Acute Leukemia

**DOI:** 10.1155/2022/1384471

**Published:** 2022-03-30

**Authors:** Li Xue, Shulan Shi, Hongtao Lei, Zhen Zhang, Xin Tian, Lijuan Qiu, Jiaolian Tang, Liyue Kui, Weiwei Nan, Xiaoyue Cao, Qiangming Sun, Ming Yu, Hongchao Jiang

**Affiliations:** ^1^Institute of Pediatrics, The Kunming Children's Hospital, Kunming, Yunnan 650228, China; ^2^Institute of Medicine, Dali University, Dali, Yunnan 671000, China; ^3^Yunnan Key Laboratory of Children's Major Disease Research, Kunming, Yunnan 650228, China; ^4^Yunnan Key Laboratory of Stem Cell and Regenerative Medicine, Biomedical Engineering Research Center, Kunming Medical University, Kunming, Yunnan 650500, China; ^5^Institute of Medical Biology, Chinese Academy of Medical Sciences and Peking Union Medical College, Kunming, Yunnan 650118, China; ^6^Yunnan Key Laboratory of Vaccine Research & Development on Severe Infectious Diseases, Kunming, Yunnan 650118, China

## Abstract

Semaphorin 4D (Sema4D) is highly expressed in various cancers and leukemia. It is involved in the development of acute leukemia. A high level of soluble Sema4D is also present in the plasma of acute leukemia patients. However, it remains unknown whether Sema4D is associated with the clinical characteristics of acute leukemia. In this study, Sema4D expression was examined in peripheral blood mononuclear cells (PBMCs) and bone marrow mononuclear cells (BMMCs) of patients with acute leukemia, and it was highly expressed in the PBMCs of B-acute lymphoblastic leukemia (ALL), T-ALL, and acute myeloid leukemia (AML) patients and in the BMMCs of B-ALL and AML patients but not in the BMMCs of T-ALL patients. Sema4D expression was higher in the PBMCs of T-ALL patients than in the PBMCs of B-ALL or AML patients. In addition, Sema4D expression in BMMCs was reduced in B-ALL patients during the chemotherapy process. It was lower in remission patients than in newly diagnosed and patients without remission. In acute leukemia, soluble Sema4D level in serum is positively correlated with Sema4D expression in PBMCs, leukocyte number, and peripheral blast number. Those results suggest that the levels of Sema4D and its soluble form are associated with acute leukemia development and may be regarded as a potential biomarker in pediatric acute leukemia.

## 1. Introduction

Pediatric acute leukemia, which includes acute myeloid leukemia (AML) and acute lymphoblastic leukemia (ALL), is the most common hematological malignancy in children and has the highest incidence in children aged 1-5 years [1], in which ALL accounts for 75-80% of pediatric acute leukemia [2]. ALL is also categorized into B cell acute lymphoblastic leukemia (B-ALL) and T cell acute lymphoblastic leukemia (T-ALL) according to the cell origin, where B-ALL accounts for approximately 85% of ALL cases [3]. Most patients treated with chemotherapy find relief after initial treatment; however, drug resistance, relapse, and extramedullary infiltration are encountered in some patients. Therefore, the search for new targets for the diagnosis and treatment of acute leukemia remains essential.

Semaphorin 4D protein (Sema4D), also known as CD100, is expressed in T lymphocytes, B lymphocytes, dendritic cells, macrophages, neutrophils, and eosinophils [4–7]. It is expressed as a transmembrane protein, which can be cleaved by matrix metalloproteinase to release soluble Sema4D from cells [8, 9]. Both forms of Sema4D participate in signal transduction, immune response, and angiogenesis regulation [10]. Sema4D is involved in autoimmune diseases, such as multiple sclerosis and rheumatoid arthritis [11]. It inhibits dendritic cell activation through interaction with Plexin-B2 in psoriasis [7] and promotes Th17 cell differentiation while inhibiting Treg cell differentiation in ankylosing spondylitis [12]. It also suppresses neutrophil activation in vasculitis [5].

Due to its important role in inflammation, Sema4D is also involved in cancer progression. It is highly expressed in breast cancer [13], pancreatic cancer [14], bladder cancer [15], head and neck squamous cell carcinoma [16], colon cancer [17], oral squamous cell carcinoma [18], ovarian cancer [19], gastric carcinoma [20], T cell non-Hodgkin's lymphomas [21], and chronic lymphoblastic leukemia [22, 23]. Sema4D promotes the proliferation and metastasis of cancer cells [15, 18, 24, 25]. Sema4D is also involved in immune response by interacting with the tumor microenvironment; soluble Sema4D level has been found to increase when coculturing with tumor-associated macrophages in a gastric carcinoma cell line [20]; the function of CD8+ T cell is inhibited when it fails to release soluble Sema4D in non-small-cell lung cancer [26]; in addition, inhibition of myeloid-derived suppressor cells is associated with Sema4D secretion in human head and neck squamous cell carcinoma [16]. As soluble Sema4D plays an important role in interacting with the tumor microenvironment, its levels in plasma are regarded as a biomarker of poor prognosis in head and neck squamous cell carcinoma [27].

In chronic lymphocytic leukemia, Sema4D promotes proliferation and inhibits apoptosis [22, 28], but the roles of Sema4D in acute leukemia are not fully clarified. We have previously shown that, in acute leukemia, Sema4D is highly expressed and soluble Sema4D levels in plasma are also elevated [29]. Sema4D promotes proliferation and inhibits apoptosis in acute leukemia and may be regarded as a potential biomarker for acute leukemia [29]; but it remains unknown whether Sema4D level in peripheral blood is associated with the clinical characteristics in acute leukemia and how Sema4D expression predicts the prognosis in acute leukemia.

In this study, to further *elucidate* the role of Sema4D as a biomarker in acute leukemia, we correlated its expression in acute leukemia and the level of its soluble Sema4D in serum with clinical characteristics.

## 2. Patients and Methods

### 2.1. Clinical Information and Sample Collection

The samples used in the study were collected from November 2019 to August 2020 at the Kunming Pediatric Hospital from patients that were diagnosed according to MICM (morphology, immunology, cytogenetics, and molecular biology) standards. The patients in the study were less than 18 years old, had been newly diagnosed with acute leukemia, and had no history of immune disease or other malignant diseases (including malignant blood disease), severe infection, previous surgery or trauma, secondary tumors, or the M3 type of AML. Samples were collected from 93 acute leukemia patients, 52 males and 41 females, including 71 ALL patients (58 B-ALL and 13 T-ALL patients), 22 AML patients, and 32 healthy children (as negative controls) (Table S1). Patients' information on sex, age, peripheral leukocyte count at first diagnosis, blast proportion, risk grading, fusion genes, chromosomes, and extramedullary infiltration was also collected.

Peripheral blood mononuclear cells (PBMCs) were collected from 35 ALL patients (25 B-ALL and 10 T-ALL), 13 AML patients, and 13 healthy children (Table S1). PBMCs were separated from peripheral blood using a human peripheral lymphocyte separation solution (Tianjin Haoyang Bioproducts Ltd., China). The blood samples were diluted twice with PBS, slowly placed on the separation solution, and centrifuged at 650 g for 20 min at 20-25°C. The PBMCs were collected, washed with PBS, and centrifuged at 650 g for 20 min at 20-25°C. The collected cells were used for further analysis or stored at -80°C.

Bone marrow mononuclear cells (BMMCs) were collected from 22 ALL patients (17 B-ALL and 5 T-ALL), 11 AML patients, 6 healthy children (Table S1), and patients who had been treated with chemotherapy for 15 days (7 patients), 33 days (4 patients), and 88 days (5 patients). The bone marrow was mixed with three volumes of red cell lysis solution (Beijing Solarbio, China), incubated on ice for 15 min, and centrifuged at 450 g for 10 min at 4°C. Then, the pellet was mixed with two volumes of red cell lysis solution, incubated on ice for 15 min, and centrifuged at 450 g for 10 min at 4°C. The cells were washed with PBS and collected for further study.

### 2.2. Western Blot

The PBMCs or BMMCs were lysed with RIPA buffer with PMSF, incubated on ice for 30 min, and centrifuged at 14,000 g for 10 min at 4°C; the protein concentration was determined by BCA method, and 20 *μ*g of the protein was subjected to SDS-PAGE, transferred to a PVDF membrane, and incubated with anti-CD100 (Cat: 53018, Cell Signaling Technology) at 1 : 1,000, CD72 (Cat: PA597292, Thermo Fisher) at 1 : 1,000, or anti-*β*-actin (Cat: AC026, Abclonal Technology) antibodies at 1 : 100,000 dilutions at 4°C overnight. The membrane was incubated with goat anti-rabbit (Cat: 074-1506, KPL) or goat anti-mouse (Cat: 074-1806, KPL) (both at 1 : 10,000 dilution) antibodies at a 20-25°C for 1 h after being washed thrice with TBST buffer and was then subjected to ECL (MilliporeSigma). The protein was quantified using ImageJ software v1.8.0 (National Institutes of Health).

### 2.3. ELISA

Serum samples were collected from 48 ALL patients (36 B-ALL and 12 T-ALL), 15 AML patients, and 13 healthy children (Table S1). Peripheral blood was collected in nonanticoagulant tubes and centrifuged at 650 g at 20-25°C for 15 min, and the serum was collected for Sema4D analysis or stored at -80°C for further study.

Soluble Sema4D was detected using a human CD100 ELISA kit (MyBioSource Ltd., USA) according to the manual, as has been described previously [29]. Briefly, the plate coated with anti-Sema4D antibodies was incubated with serum samples or a Sema4D standard at 37°C for 2 h and then incubated with biotin-conjugated anti-Sema4D antibodies at 37°C for 2 h. The plate was incubated with HRP-streptavidin at 37°C for 1 h after being washed thrice with the washing solution provided in the kit. Then, the plate was washed five times, incubated with 3,3′,5,5′-tetramethylbenzidine (TMB) solution for 15 min, and read at 450 nm after terminating the reaction.

### 2.4. Statistical Analysis

Statistical analysis was performed by SPSS 22.0. The normal distribution of the data was checked using the homogeneity of variance test. For data with an even variance, Student's *t*-test was performed on two groups of data, and one-way ANOVA followed by Bonferroni's post hoc test was performed on multiple groups of data. Logarithmic conversion was performed for data with an uneven variance; if the variance was even after data processing, the abovementioned analysis was performed; if the variance remained uneven after data processing, the Man-Whitney test was performed on two groups of data, and the Kruskal-Wallis test followed by Dunn's test was performed on multiple groups of data. For correlation analyses, the data normal distribution was also examined using the homogeneity of variance test; normally distributed data were performed using the Pearson method, and nonnormally distributed data were performed using the Spearman method.

## 3. Results

### 3.1. Sema4D Is Highly Expressed in PBMCs and Positively Correlated with the Soluble Sema4D Level in Serum of Acute Leukemia

**Figure 1:**
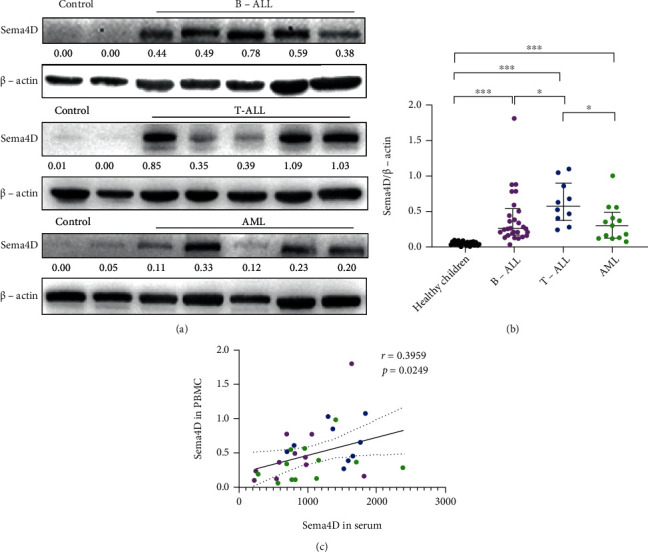
Sema4D is highly expressed in the PBMCs of patients with acute leukemia. (a) Western blot analysis of Sema4D expression in the PBMCs of B-ALL, T-ALL, and AML patients and healthy children (control). The protein level of each blot was quantified relatively to the internal control *β*-actin. (b) Quantification of Western blot analysis of Sema4D. (c) Correlation of Sema4D expression in PBMCs with the soluble Sema4D level in serum. Magenta: B-ALL, blue: T-ALL, and green: AML; ^∗^*p* < 0.05 and ^∗∗∗^*p* < 0.001.

We have previously shown that Sema4D is highly expressed in acute leukemia, and the soluble Sema4D level is increased in plasma [29]. In this study, we collected PBMCs and investigated Sema4D expression in B-ALL, T-ALL, and AML patients by Western blotting. Sema4D was highly expressed in B-ALL, T-ALL, and AML patients compared with healthy children (Figures 1(a) and 1(b)); Sema4D expression was significantly higher in T-ALL patients than in B-ALL or AML patients (Figure 1(b)). We also found that Sema4D expression in PBMCs was positively correlated with soluble Sema4D level in serum (Figure 1(c)). We further examined the expression of CD72 (the receptor of Sema4D in B lymphocytes, T lymphocytes, and myeloid cells) in PBMCs of B-ALL, T-ALL, and AML; its expression increased in B-ALL, T-All, and AML patients compared to healthy children (Supplementary Fig 1a, b and c). Consistent with our previous study [29], the expression of PI3K and ERK also increased in the PBMCs of B-ALL, T-ALL, and AML compared to healthy children (Supplementary Fig 1a, b and c).

### 3.2. Sema4D Is Highly Expressed in BMMCs of Patients with B-ALL or AML

**Figure 2:**
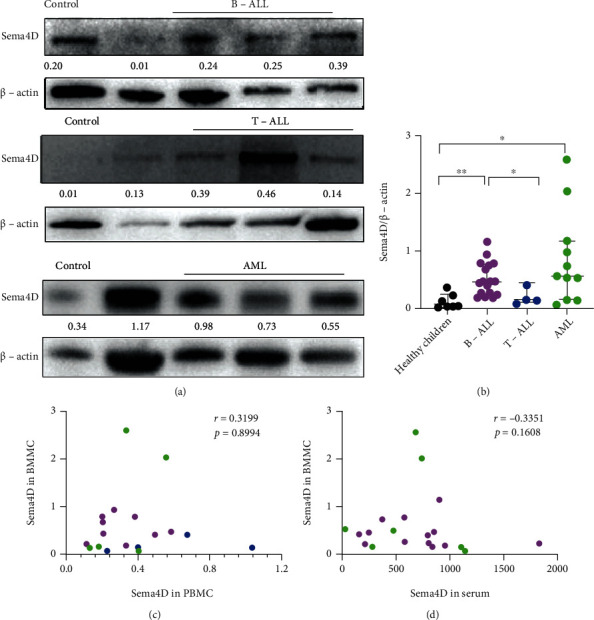
Sema4D is highly expressed in BMMCs of patients with B-ALL or AML. (a) Western blot analysis of Sema4D expression in the BMMCs of B-ALL, T-ALL, and AML patients and healthy children (control). The protein level of each blot was quantified relatively to the internal control *β*-actin. (b) Quantification of Western blot analysis. (c) Correlation of Sema4D expression in PBMCs with BMMCs. (d) Correlation of Sema4D expression in BMMCs with the soluble Sema4D level in serum. Magenta: B-ALL; blue: T-ALL; and green: AML. ^∗^*p* < 0.05 and ^∗∗^*p* < 0.01.

We further investigated the Sema4D expression in BMMCs, and Sema4D was highly expressed in B-ALL and AML patients compared with healthy children, but there was no difference in Sema4D expression between T-ALL patients and healthy children (Figures 2(a) and 2(b)). In acute leukemia, Sema4D expression in BMMCs was not correlated with Sema4D expression in PBMCs (Figure 2(c)). Considering that Sema4D expression is only highly expressed in BMMCs of AML and B-ALL (Figure 2(b)), the correlation analysis was performed on Sema4D expression in BMMCs of AML and B-ALL and the soluble Sema4D level in serum, and no correlation was found (Figure 2(d)).

### 3.3. Sema4D Levels in Serum Are Correlated with Leukocyte Number and Peripheral Blast Number in Acute Leukemia

**Figure 3:**
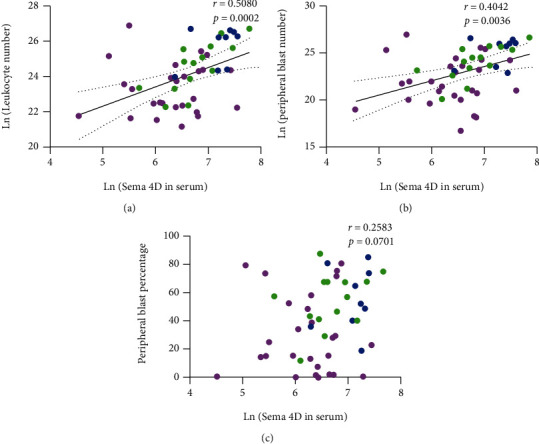
Sema4D levels in serum are correlated with leukocyte number and peripheral blast number in acute leukemia. (a) Correlation of soluble Sema4D level with leukocyte number. (b) Correlation of soluble Sema4D level with peripheral blast number. (c) Correlation of soluble Sema4D level with peripheral blast number percentage. Magenta: B-ALL; blue: T-ALL; and green: AML.

We performed a correlation analysis of Sema4D expression in PBMCs or BMMCs with age, sex, leukocyte number, peripheral blast number, risk classification, early remission status, extramedullary infiltration, chromosome translocation, and gene fusion. Sema4D expression in PBMCs of ALL and AML patients and BMMCs of ALL patients was not correlated with risk classification, early remission status, extramedullary infiltration, chromosome translocation, and fusion genes (Tables S2-4). As there were insufficient BMMC samples obtained from AML patients, we did not analyze the correlation of Sema4D expression in BMMCs with clinical characteristics in AML. We also performed a correlation analysis of the soluble Sema4D level in serum with clinical characteristics, and the data showed that the soluble Sema4D was positively correlated with the leukocyte number (Figure 3(a)) and peripheral blast number (Figure 3(b)), but not with the percentage of peripheral blasts (Figure 3(c)).

### 3.4. Sema4D Expression Is Reduced in B-ALL Patients during the Chemotherapy Process

**Figure 4:**
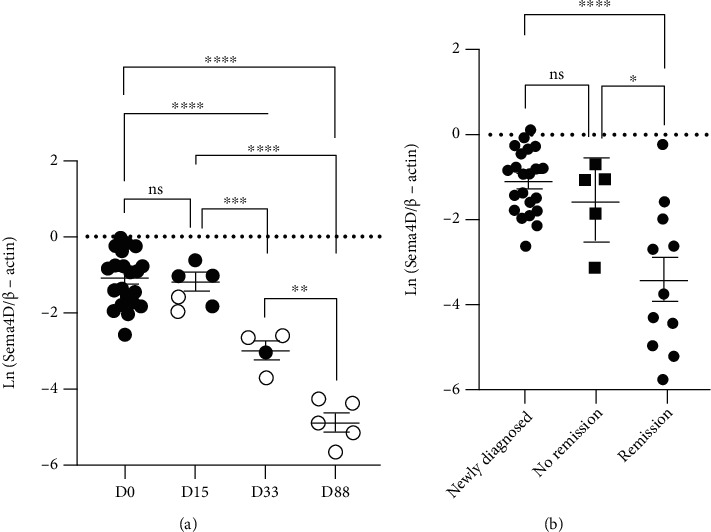
Sema4D expression is reduced in B-ALL patients during the chemotherapy process. (a) Comparison of Sema4D expression in the BMMCs of newly diagnosed patients (D0) and patients who received chemotherapy for 15 (D15), 33 (D33), and 88 days (D88); solid dots: no remission; empty dots: remission. (b) Comparison of Sema4D expression in BMMCs among newly diagnosed patients, patients without remission, and remission patients. ^∗^*p* < 0.05, ^∗∗^*p* < 0.01, ^∗∗∗^*p* < 0.001, and ^∗∗∗∗^*p* < 0.0001; ns: not significant.

The Sema4D expression in BMMCs was examined in B-ALL patients who had received chemotherapy, and the PBMCs were not collected during the chemotherapy for the ethical consideration. ALL was diagnosed based on the standard procedure, and the corresponding chemotherapy regimens were followed. The data showed no difference in Sema4D expression between newly diagnosed patients (D0) and patients who had received 15 days of treatment (D15) (Figure 4(a)). There was a significant difference in Sema4D expression between D0 and D33 or D88, between D15 and D33 or D88, and between D33 and D88 (Figure 4(a)). Sema4D expression in remission patients was found to be significantly lower than in newly diagnosed patients and patients without remission (Figure 4(b)). The results suggested that chemotherapy and the remission process reduced Sema4D expression.

## 4. Discussion

Sema4D is highly expressed in various cancer cells [13, 15–17, 30]. It promotes proliferation, angiogenesis, and metastasis and inhibits apoptosis in tumors. We previously found that Sema4D is highly expressed in pediatric acute leukemia, which is a serious threat to children and one of the main causes of death in children; Sema4D expression is also correlated with the phosphorylation of PI3K, ERK, and AKT in PBMCs of acute leukemia patients [29]. Our study with overexpression and knockdown of Sema4D in acute leukemia cell lines showed that Sema4D promotes proliferation and inhibits apoptosis in acute leukemia by activating the PI3K/AKT and ERK signaling pathways [29].

To explore whether Sema4D is a clinically potential biomarker in acute leukemia, we investigated the correlation of Sema4D expression in PBMCs and BMMCs with clinical characteristics in this study. Our data suggested that Sema4D expression was high in PBMCs of T-ALL, B-ALL, and AML patients, while Sema4D expression in PBMCs of T-ALL patients was even higher than that of B-ALL or AML patients. Children with T-ALL often have a poor prognosis [31], in which Sema4D may be an important factor.

Sema4D was highly expressed in BMMCs of B-ALL and AML patients, but not in BMMCs of T-ALL patients. As precursors of T lymphocytes develop in the thymus and infiltrate into the bone marrow [32] and B lymphocytes or myeloid cells mainly develop in the bone marrow, it is not surprising that Sema4D expression is lower in BMMCs of T-ALL patients than in the BMMCs of B-ALL or AML patients.

Sema4D expression in BMMCs was reduced in ALL patients following chemotherapy and remission; as patients' initial response to chemotherapy is an important indicator of prognosis, it may be an indicator for assessing treatment efficacy in children with ALL.

Sema4D level is not correlated with risk classification and early remission status, suggesting it may not be the main drive force in acute leukemia development and is not involved in the relapse. Sema4D may also not be involved in the process of extramedullary infiltration, chromosome translocation, and fusion genes, as it is also not correlated with those clinical characteristics.

Soluble Sema4D is released by proteolytic cleavage of the transmembrane form of Sema4D and circulates in the blood, which interacts with the tumor microenvironment. It interacts with T cells and B cells by binding CD72 [11], interacts with endothelial cells by binding PlexinB1 [33], and activates endothelial differentiation through the ERK and AKT pathways [34]. Soluble Sema4D levels in plasma may be regarded as a biomarker in head and neck squamous cell carcinoma [35], and high levels of soluble Sema4D are associated with the INF-*γ*-negative tumor microenvironment [27]. Our previous study also showed high soluble Sema4D levels in the blood [29], and in this study, we found that soluble Sema4D is positively correlated with Sema4D expression in PBMCs but not in BMMCs, as the serum was collected from peripheral blood, not from bone marrow, it is not surprising soluble Sema4D is only correlated with Sema4D expression in PBMCs. Soluble Sema4D is also positively correlated with the leukocyte number and peripheral blast number. As blasts mainly exist in the bone marrow in healthy people but not in peripheral blood, high Sema4D levels in serum may indicate infiltration and proliferation of leukemia cells in peripheral blood. Acute leukemia development may be monitored by ELISA detection of soluble Sema4D, which is easier and more convenient than traditional methods. As Sema4D is also expressed in other tumors and may also be released into blood, whether those tumors could also be monitored by soluble Sema4D detection remains to be studied.

## 5. Conclusions

Our study showed high Sema4D expression in both PBMCs and BMMCs of B-ALL and AML and in PBMCs of T-ALL. The soluble Sema4D level in serum is positively correlated with Sema4D expression in PBMCs, leukocyte number, and peripheral blast number, suggesting that soluble Sema4D may be regarded as a potential biomarker for pediatric acute leukemia development and prognosis. The function of soluble Sema4D in acute leukemia will be investigated in a future study.

## Data Availability

All data generated or analyzed during this study are included in this published article.

## Supplementary Materials

Supplementary 1 Supplementary Figure 1: the expression of CD72, PI3K, and ERK increased in PBMCs of acute leukemia patients. (a) Western blot analysis of CD72, PI3K, and ERK expression in PBMCs of B-ALL patients. (b) Western blot analysis of CD72, PI3K, and ERK expression in PBMCs of T-ALL patients. (c) Western blot analysis of CD72, PI3K, and ERK expression in PBMCs of AML patients. The protein level of each blot was quantified relatively to the internal control *β*-actin.

Supplementary 2 Table S1: details of collected samples in the study. Table S2: analysis of Sema4D expression in PBMCs with clinical characteristics in ALL. Table S3: analysis of Sema4D expression in PBMCs with clinical characteristics in AML. Table S4: analysis of Sema4D expression in BMMCs with clinical characteristics in ALL.
