# Supplementary figures and images for: Soluble Sema4D Level Is Positively Correlated with Sema4D Expression in PBMCs and Peripheral Blast Number in Acute Leukemia
Source: Dis Markers. 2022 Mar 30;2022:1384471. doi: 10.1155/2022/1384471 (PMC8988092; doi:10.1155/2022/1384471)

Supplementary Figure 1

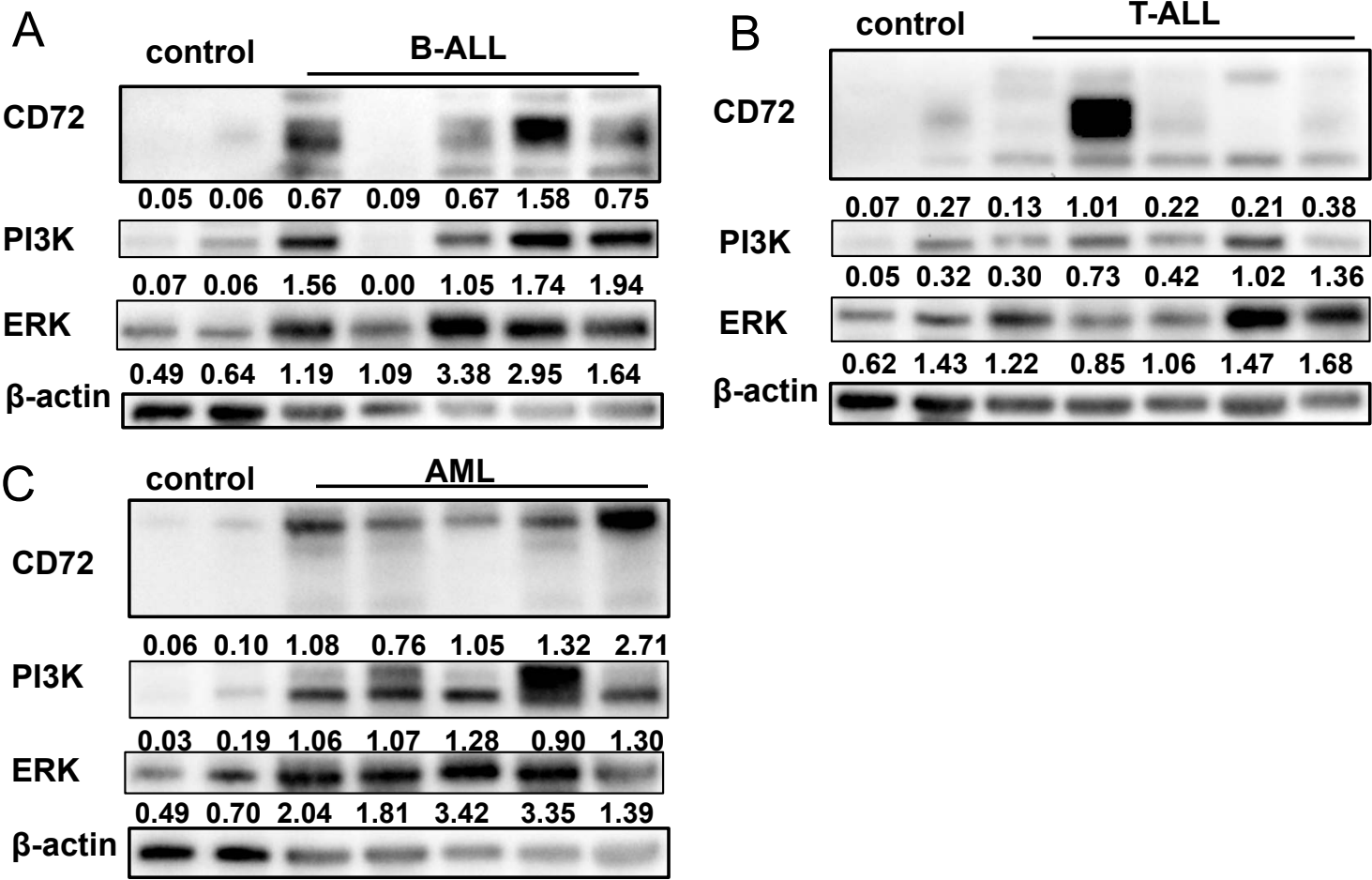

Supplement: Supplementary 1 — Supplementary Figure 1: the expression of CD72, PI3K, and ERK increased in PBMCs of acute leukemia patients. (a) Western blot analysis of CD72, PI3K, and ERK expression in PBMCs of B-ALL patients. (b) Western blot analysis of CD72, PI3K, and ERK expression in PBMCs of T-ALL patients. (c) Western blot analysis of CD72, PI3K, and ERK expression in PBMCs of AML patients. The protein level of each blot was quantified relatively to the internal control β-actin. [file 1384471.f1.pdf]
