# Supplementary material for: Soluble Sema4D Level Is Positively Correlated with Sema4D Expression in PBMCs and Peripheral Blast Number in Acute Leukemia
Source: Dis Markers. 2022 Mar 30;2022:1384471. doi: 10.1155/2022/1384471 (PMC8988092; doi:10.1155/2022/1384471)
Supplement: Supplementary 2 — Table S1: details of collected samples in the study. Table S2: analysis of Sema4D expression in PBMCs with clinical characteristics in ALL. Table S3: analysis of Sema4D expression in PBMCs with clinical characteristics in AML. Table S4: analysis of Sema4D expression in BMMCs with clinical characteristics in ALL. [file 1384471.f2.docx]

**Supplementary Table**

**Table S1. Details of collected samples in the study**

|  | B-ALL | T-ALL | AML | Healthy children |
| --- | --- | --- | --- | --- |
| PBMCs+BMMCs+Serum | 2 | 3 | 4 | - |
| PBMCs+BMMCs | 4 | 1 | 1 | - |
| PBMCs+Serum | 6 | 6 | 7 | - |
| BMMCs+Serum | 6 | 1 | 1 | - |
| PBMCs only | 13 | - | 1 | 13 |
| BMMCs only | 5 | - | 5 | 6 |
| Serum only | 22 | 2 | 3 | 13 |
| Total | 58 | 13 | 22 | 32 |

ALL: acute lymphoblastic leukemia; AML: acute myeloid leukemia; PBMCs: peripheral blood mononuclear cells; BMMCs: bone marrow mononuclear cells.

**Table S2．** **Analysis of Sema4D expression in PBMCs with clinical characteristics in ALL**

| Characteristic | ALL(n) | Sema4D expression | P value |
| --- | --- | --- | --- |
| Total | 34 |  |  |
| Gender |  |  | 0.822 |
| male | 19 | 0.38(0.20,0.78) |  |
| female | 15 | 0.33(0.20,0.59) |  |
| Age (years) |  |  | 0.380 |
| 1-10 | 25 | 0.33(0.16,0.60) |  |
| <1 or ≥10 | 9 | 0.35(0.23,0.81) |  |
| leukecyte count（×10^9^/L） |  |  | 0.285 |
| <50 | 16 | 0.30(0.15,0.51) |  |
| ≥50 | 18 | 0.41(0.22,0.70) |  |
| Risk stratification |  |  | 0.380 |
| SR+IR | 17 | 0.40(0.18,0.78) |  |
| HR | 17 | 0.28(0.21,0.48) |  |
| D15-Remission |  |  | 0.149 |
| Yes | 12 | 0.46(0.21,0.85) |  |
| No | 22 | 0.27(0.16,0.52) |  |
| D33-Remission* |  |  | 0.354 |
| Yes | 21 | 0.38(0.18,0.81) |  |
| No | 9 | 0.27(0.18,0.40) |  |
| Extramedullary infiltration |  |  | 0.678 |
| Yes | 24 | 0.30(0.20,0.74) |  |
| No | 10 | 0.37(0.15,0.54) |  |
| Karyotype |  |  | 0.496 |
| Normal | 24 | 0.34(0.20,0.75) |  |
| Abnormal | 10 | 0.33(0.14,0.52) |  |
| Fusion gene |  |  |  |
| E2A/PBX1（+） | 4 | 0.46(0.18,0.73) | 0.831 |
| E2A/PBX1（-） | 30 | 0.32(0.20,0.63) |  |
| BCR-ABL（+） | 3 | 0.23(0.22,0.23) | 0.316 |
| BCR-ABL（-） | 31 | 0.38(0.20,0.67) |  |
| WT1（+） | 4 | 0..30(0.13,0.74) | 0.630 |
| WT1（-） | 30 | 0.34(0.20,0.63) |  |

***** Data of three samples was not collected

**Table S3．** **Analysis of Sema4D expression in PBMCs with clinical characteristics in AML**

| Characteristic | AML(n) | Sema4D expression | P value |
| --- | --- | --- | --- |
| Total | 13 |  |  |
| Gender |  |  | 0.866 |
| male | 10 | 0.23(0.12,0.55) |  |
| female | 3 | 0.36(0.06,0.36?) |  |
| Age (years) |  |  | 0.286 |
| 1-10 | 7 | 0.25±0.188 |  |
| <1 or ≥10 | 6 | 0.41±0.322 |  |
| leukocyte count（×10^9^/L） |  |  | 0.501 |
| <100 | 5 | 0.26±0.209 |  |
| ≥100 | 8 | 0.37±0.294 |  |
| Immunophenotype |  |  | 0.350 |
| M2 | 7 | 0.26±0.180 |  |
| Others | 6 | 0.40±0.33 |  |
| Risk stratification |  |  | 0.265 |
| SR+IR | 5 | 0.22±0.120 |  |
| HR | 8 | 0.39±0.309 |  |
| D21/28-Remission |  |  | 0.093 |
| Yes | 7 | 0.24±0.178 |  |
| No | 4 | 0.54±0.346 |  |
| Extramedullary infiltration |  |  | 0.214 |
| Yes | 6 | 0.23±0.136 |  |
| No | 7 | 0.41±0.320 |  |
| Karyotype |  |  | 0.315 |
| Normal | 7 | 0.40±0.305 |  |
| Abnormal | 6 | 0.25±0.191 |  |
| Fusion gene |  |  |  |
| AML1-ETO（+） | 4 | 0.27(0.09,0.39) | 0.758 |
| AML1-ETO（-） | 9 | 0.29(0.12,0.56) |  |
| WT1（+） | 6 | 0.27(0.10,0.55) | 0.886 |
| WT1（-） | 7 | 0.29(0.13,0.40) |  |

* Data of two samples was not collected.

**Table S4.** **Analysis of Sema4D expression in BMMCs with clinical** **characteristics in ALL**

| Characteristic | ALL(n) | Sema4D expression | P value |
| --- | --- | --- | --- |
| Total | 17 |  |  |
| Gender |  |  | 0.922 |
| male | 10 | 0.31(0.14,0.75) |  |
| female | 7 | 0.24(0.17,0.77) |  |
| Age (years) |  |  | 0.910 |
| 1-10 | 13 | 0.24(0.16,0.76) |  |
| <1 or ≥10 | 4 | 0.41(0.10,1.03) |  |
| leukocyte count（×10^9^/L） |  |  | 0.688 |
| <50 | 11 | 0.23(0.15,0.74) |  |
| ≥50 | 6 | 0.52(0.14,0.77) |  |
| Risk stratification |  |  | 0.315 |
| SR+IR | 11 | 0.23(0.15,0.74) |  |
| HR | 6 | 0.52(0.17,0.99) |  |
| D15-Remission |  |  | 0.246 |
| Yes | 5 | 0.17(0.14,0.51) |  |
| No | 12 | 0.32(0.18,0.77) |  |
| D33-Remission |  |  | 0.140 |
| Yes | 12 | 0.20(0.14,0.72) |  |
| No | 5 | 0.25(0.22,1.04) |  |
| Extramedullary infiltration |  |  | 0.696 |
| Yes | 10 | 0.22(0.16,0.75) |  |
| No | 7 | 0.25(0.15,0.77) |  |
| Karyotype |  |  | 0.763 |
| Normal | 11 | 0.24(0.17,0.66) |  |
| Abnormal | 6 | 0.47(0.13,0.99) |  |
| Fusion gene |  |  |  |
| E2A/PBX1（+） | 1 | 0.17 | / |
| E2A/PBX1（-） | 16 | 0.25(0.15,0.76) |  |
| BCR-ABL（+） | 3 | 0.66(0.08,0.90) | 0.705 |
| BCR-ABL（-） | 14 | 0.24(0.16,0.75) |  |
| WT1（+） | 4 | 0.23(0.16,0.93) | 0.910 |
| WT1（-） | 13 | 0.24(0.16,0.76) |  |
